# Human brain data: a valuable and open-ended resource for artificial intelligence

**DOI:** 10.3389/fnhum.2026.1808115

**Published:** 2026-04-28

**Authors:** Maël Donoso

**Affiliations:** Ouroboros Neurotechnologies, Lausanne, Switzerland

**Keywords:** AI safety, alignment, brain foundation models, brain-trained AI, brain-trained foundation models, NeuroAI, neuroimaging, reasoning

## Abstract

Experimental evidence increasingly demonstrates that human brain data can be leveraged to train artificial intelligence models. In this context, neuroimaging data is both valuable, since it can improve model performance and could be prioritized for high-value steps in model training, and open-ended, since future neuroscience discoveries could retroactively identify new neural signals of interest in current neuroimaging datasets. The emergence of human brain data as a valuable and open-ended resource for artificial intelligence, including for foundation models, could translate into a new and strategic role for neuroscientists, and open new possibilities for the simulation of human cognition.

## 1 Introduction

When neuroscientists acquire neuroimaging data on human subjects, their objective is usually to understand the functioning of the human brain in a particular context. To achieve this objective, they typically design a neuroscience experiment eliciting the cognitive processes of interest, then rely on a variety of neuroimaging technologies such as functional magnetic resonance imaging (functional MRI, or fMRI), electroencephalography (EEG), or magnetoencephalography (MEG) to uncover the neural correlates of these processes. Even when human brain data is acquired to test specific hypotheses about human cognition, neuroimaging datasets can often be repurposed for other analyses, and used to extract new neural signals of interest as neuroscience theories evolve.

Remarkably, a growing number of studies demonstrate that neuroimaging data can also be repurposed for a different objective: improving the performance of classical machine learning, deep learning, or reinforcement learning models for a variety of perceptual, executive, or semantic tasks. Experimental evidence suggests that training models on human brain data could help artificial intelligence (AI) systems to better approximate some elements of human cognition, and open a promising path to overcome some of their current limitations. Here, we use the word “training” in an inclusive sense, encompassing any learning, regularization, fine-tuning, or alignment method that leverages neuroimaging data to improve the performance of a model. Regardless of the specific algorithm, this emerging approach could represent a significant conceptual shift at the intersection of neuroscience and AI, complementing the classical paradigm of *brain-inspired AI* (designing AI models inspired by neuroscience discoveries) with the new possibilities of *brain-trained AI* (training AI models directly on neuroimaging data).

As advances in AI continue to exert a profound influence on neuroscience research, this conceptual shift could provide an opportunity to renew and strengthen the reciprocal impact from neuroscience to AI. Indeed, while general-purpose foundation models such as large language models (LLMs) have achieved impressive results across a variety of domains, the information that can be extracted from semantic data alone, such as text, might become a limiting factor as a large fraction of the available data has already been used for model training, and as the continued scaling of training datasets seems to yield diminishing returns. Furthermore, this limitation might extend beyond LLMs, since even in large multimodal models (LMMs), linguistic content such as annotations plays a critical role by acting as a semantic interface between different data modalities. When relevant, complementing such semantic data with neural signals could emerge as an original strategy for leveraging more directly the hidden variables of human brain activity, and not just the behavioral products of human cognition. A theoretical foundation for this strategy, from an AI perspective and focusing on high-level formalization and methods, is proposed elsewhere (Donoso, 2026). In this paper, we focus instead on the status of human brain data as an increasingly valuable and distinctively open-ended training resource, and on the role of neuroscientists as the curators of this resource.

**Figure 1:**
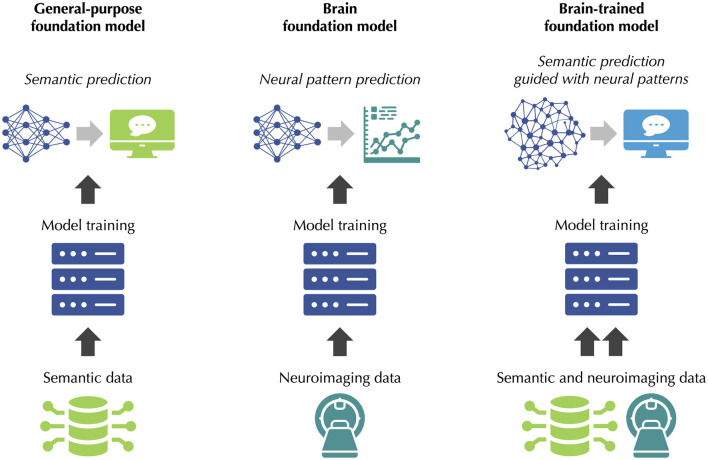
Brain-trained foundation models. **Left:** General-purpose foundation models, such as LLMs, are trained on semantic data, such as text. They are used for semantic prediction. **Middle:** Brain foundation models are trained on neuroimaging data, such as MRI or EEG data. They are used for neural pattern prediction. **Right:** Hypothetical brain-trained foundation models would be trained on both semantic and neuroimaging data. Their objective would be to improve the performance of general-purpose foundation models by guiding semantic prediction with neural patterns.

Hypothetical *brain-trained foundation models* should be distinguished from the large AI models currently developed for neuroscience and neurotechnology, which are sometimes known as *brain foundation models* but are not our primary focus here, as illustrated in Figure 1. The objective of current brain foundation models, which are typically pre-trained on an extensive corpus of neuroimaging data, such as MRI (Tak et al., 2026) or EEG data (Ouahidi et al., 2025), is to predict neural patterns within these neuroimaging modalities. By contrast, the objective of hypothetical brain-trained foundation models would be to leverage neuroimaging data to improve the performance of general-purpose foundation models, such as LLMs or LMMs, on the wide range of tasks for which these models are used. Fundamentally, the hypothesis behind brain-trained foundation models is that neural signals could help LLMs or LMMs to move beyond surface-level statistical regularities, by complementing the knowledge gained from semantic data with the deeper insights obtained directly from the human brain. While such a strategy is not yet part of the training pipelines of foundation models, it has already proved successful in a variety of narrower, smaller-scale settings, which increasingly overlap with the functions of LLMs or LMMs, as we will now summarize.

## 2 A valuable resource

Existing research on improving the performance of AI models using neuroimaging data has often focused on perception. In the context of object recognition, foundational studies have shown that fMRI data can be leveraged to improve classical machine learning models (Fong et al., 2018), and EEG data to improve deep learning models (Spampinato et al., 2017), while also suggesting that such methods could be extended to other neuroimaging technologies, learning algorithms, or sensory modalities. Other studies have continued to explore how deep learning models for visual tasks can be guided using EEG data (Hansen et al., 2025), and extended the investigation to auditory tasks using fMRI data (Freteault et al., 2025). There is also evidence that reward and error signals extracted from EEG data can be used to guide reinforcement learning models in robotics (Iturrate et al., 2010; Kim et al., 2017), demonstrating that the valuation processes of the human brain can be leveraged to improve the performance of AI models on executive tasks. Finally, despite the distributed and intricate nature of the networks associated with symbolic thought and expression in the human brain, studies have used fMRI or MEG data to align language models with human semantic representations (Schwartz et al., 2019), and to improve the performance of auditory models on semantic tasks (Moussa et al., 2025).

The common motivation behind these studies is the intuition that human brain activity, as approximated by neuroimaging data, can provide valuable insights for training AI models, beyond what is already accessible through the behavioral products of human cognition such as text, image annotations, or sound annotations. The performance improvements reported in these experiments are non-trivial, since they demonstrate that a relatively low signal-to-noise ratio (SNR), high individual variation, and other challenging characteristics often encountered in neuroimaging data are not an insurmountable obstacle for leveraging neural signals in the context of AI model training. Interestingly, existing research has reported model improvement with as few as eight human subjects (Moussa et al., 2025), suggesting that neuroimaging datasets might be a valuable training resource even when the number of participants is limited, provided that enough data is acquired for each subject. The relatively low SNR in neuroimaging data can occasionally be challenging, particularly when models are trained on individual subjects (Freteault et al., 2025). However, the effects of noise can be mitigated, for example, by performing sample selection (Fong et al., 2018) or estimating the noise ceiling for each voxel (Moussa et al., 2025) in fMRI studies, or by applying spatial filtering (Kim et al., 2017) or performing electrode selection (Hansen et al., 2025) in EEG studies.

The question of whether such efforts can be scaled is legitimate. While existing research demonstrates the feasibility of brain-trained AI, experimental evidence remains relatively limited in both scale and task diversity. By contrast, foundation models such as LLMs have emerged as general-purpose systems trained on large-scale data, and deployable across a wide range of applications. This naturally invites reflection on the size and diversity of neuroimaging datasets that would be necessary to meaningfully complement the more classical training data types of foundation models. Still, it seems reasonable to assume that the depth of human brain data, rather than its quantity, would probably be its most valuable characteristic from the perspective of foundation model training. Whereas classical training data relies on the behavioral outputs of human cognition, neuroimaging data could open a window into the latent cognitive processes that generated these outputs in the first place, potentially providing unique insights for improving the robustness and generality of the models. A promising path could also be to prioritize the use of human brain data for strategically chosen, high-value steps in foundation model training, where the limitations of classical training data are the most apparent, as we will now discuss.

## 3 An open-ended resource

Foundation model training usually follows a three-step process: pre-training; supervised fine-tuning; and preference fine-tuning, typically using reinforcement learning from human feedback (RLHF) to align the model with human values. The third step presents specific challenges, as the model must infer general human preferences from a limited set of surface-level information, such as the ratings provided by the annotators. As a result, foundation models remain imperfectly aligned with human values, particularly in novel or ambiguous contexts (Khamassi et al., 2024). Here, it seems natural to envision an extension of RLHF in which neuroimaging data could be leveraged for a deeper representation of human preferences (Donoso, 2026). In particular, the neural signals associated with reward, value, confidence, surprise, effort, or conflict, which are well-characterized in affective neuroscience, could provide unique insights into the latent cognitive processes of human valuation. Importantly, RLHF is already an expensive step, suggesting that the addition of at least some neuroimaging modalities, such as EEG, could be realistic in economic terms. Other neuroimaging modalities such as fMRI or MEG would result in higher costs, although the neural signals extracted from the data could also be more comprehensive. Ethical considerations regarding the use of human brain data for foundation model training are briefly discussed in the next section.

Beyond alignment, another strategic use for neuroimaging data could be reasoning. At inference time, foundation models are expected to engage in multi-step inference, using techniques such as chain-of-thought (CoT) prompting. Implementing such capabilities is particularly challenging, as the model must approximate human executive function from surface-level examples of human reasoning. As a result, foundation models remain limited in their reasoning abilities, and strategies to expand multi-step inference typically come at the expense of a greater computational cost (Besta et al., 2024). It has been famously argued that deep learning needs a prefrontal cortex (Russin et al., 2020), but perhaps we could start taking this argument more literally, and envision an extension of the CoT approach in which neuroimaging data could be leveraged for a better approximation of human executive function (Donoso, 2026). In particular, the neural signals associated with task switching, the reliabilities of current and alternative task sets, and the value of exploratory behavior, which are well-characterized in decision neuroscience, could provide unique insights into the latent cognitive processes of human multi-step thinking. Guiding model reasoning with human brain data may not be as straightforward as guiding model alignment, since neuroimaging data can typically only be acquired over relatively short periods, whereas foundation models are often expected to reason over longer problems. However, this temporal mismatch may not be a fundamental obstacle for extracting useful patterns from neuroimaging data, as long reasoning sequences may be decomposable into shorter tasks, compatible with the timescales of fMRI, EEG, or MEG acquisition.

**Figure 2:**
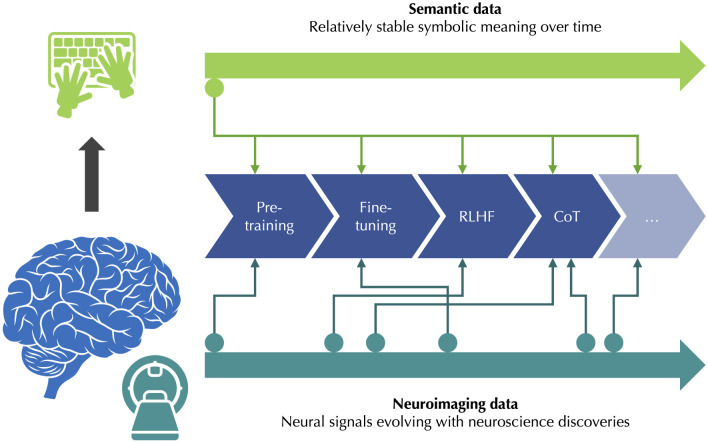
Human brain data as a valuable and open-ended resource. **Left:** Human brain activity can be approximated by neuroimaging data, while being the source of semantic data such as text. **Right:** Semantic data has a relatively stable symbolic meaning over time; once acquired, it can be directly used for the intended steps in AI model training. By contrast, neuroimaging data allows for the extraction of neural signals evolving with neuroscience discoveries; once acquired, it can be reinterpreted over time, and potentially applied to previously unexpected steps in AI model training.

Regardless of the specific algorithms, alignment and reasoning could be promising targets in the attempt to prioritize the use of human brain data for strategically chosen, high-value steps in foundation model training. While dedicated neuroimaging datasets may be acquired specifically for this objective, it seems also realistic to think that in some cases, existing datasets in fields such as affective or decision neuroscience may be repurposed to extract the neural signals of interest, at least for exploration or calibration experiments. Indeed, a single neuroimaging dataset can simultaneously contain information on a diversity of cognitive processes such as perception, valuation, memory, and executive control, even if only one of these processes is explicitly manipulated by the experimental design. The degree of open-endedness of human brain data is not new to neuroscience, but it would be relatively new and distinctive in the context of foundation model training, where classical semantic data such as text, image annotations, or sound annotations has a more stable symbolic meaning. Arguably, neuroimaging datasets might even be among the few dataset types combining both a high degree of open-endedness and a true semantic relevance, since they reflect our evolving understanding of human cognition, whereas natural images or sounds, for example, may be reinterpreted over time but are not a direct semantic product of the human brain. As future neuroscience discoveries could retroactively identify new neural signals of interest in current neuroimaging datasets, human brain data could emerge as an increasingly relevant resource for AI model training, and perhaps hold the keys for overcoming the future limitations of foundation models, as illustrated in Figure 2.

## 4 Challenges and opportunities

Neuroscientists are already well aware of the ethical and social challenges associated with human brain data, and experienced in navigating the consent, privacy, and anonymity constraints of neuroimaging datasets. If these datasets were extensively leveraged for foundation model training, it seems reasonable to assume that the rules governing data acquisition could be reevaluated, and possibly strengthened, for example with additional protections or clarifications regarding the security and ownership of the data. However, before diving into the procedural details, neuroscientists may need to answer a larger strategic question: whether or not they embrace this new objective of neuroimaging data, and if they do, to what extent and under which conditions. Indeed, brain-trained AI has the potential to redefine the role of human cognitive and computational neuroscience, complementing its primary mission of understanding the human brain with the secondary objective of co-developing AI models. The decision of the neuroscience community on this matter could have a profound impact on the future, as its collective influence could either accelerate, decelerate, or reorient the development of brain-trained AI. Making this decision would certainly require taking into consideration the risks, such as the possibility that the neural signals could be used to predict individual human actions at scale, but also the opportunities, such as the potential for improving the alignment with human values and the reasoning capabilities of foundation models. These opportunities could be particularly significant from the perspective of AI safety, as the development of neurally aligned AI might open an original path toward a solution to the alignment problem.

Assuming that neuroscientists decide to embrace the perspective of brain-trained AI, some technical requirements would likely need to be met in order to facilitate the repurposing of neuroimaging data for this objective. Foundation model training would certainly benefit from well-annotated, standardized neuroimaging datasets, in line with the current efforts of the neuroscience community to improve the quality and reproducibility of human brain research. Although the acquisition of large-scale homogeneous datasets, focusing on a single task such as image recognition, may be useful for improving a specific function of foundation models, brain-trained AI could also benefit from a diversity of neuroscience research programs spanning multiple populations, cognitive functions, experimental designs, and neuroimaging modalities, as such diversity would increase the open-ended value of human brain data. If dedicated neuroimaging datasets are acquired for brain-trained AI, these requirements suggest the possibility of complementary roles for AI companies and neuroscience laboratories according to their respective strengths, with the former potentially leading the acquisition of large-scale homogeneous datasets, and the latter focusing more on fundamental research and smaller-scale experiments. Such an arrangement could be mutually beneficial, provided that a significant fraction of the acquired datasets are publicly released on both sides. Additionally, brain-trained foundation models would probably require complex pipelines for processing large-scale neuroimaging data, extracting the relevant neural signals, and leveraging these signals in the context of foundation model training. An interesting possibility could be to reuse some of the methods developed for brain foundation models, which already address the challenge of processing neuroimaging data at scale, and adapt these methods to the specificities of brain-trained foundation models. Furthermore, while existing research on improving the performance of AI models using neuroimaging data typically focuses on a single modality, such as fMRI, EEG, or MEG, it seems also possible to envision brain-trained foundation models jointly guided by different types of neural signals. Such a multimodal training strategy could add a new layer of complexity, in particular because different neuroimaging techniques imply different spatial scales and temporal dynamics, but the methods developed for multimodal experiments, such as EEG-fMRI studies, could help to address these additional technical requirements.

Together, these technical challenges and opportunities could open new horizons regarding the feasibility of brain-trained AI. Existing studies may be relatively limited in scale and task diversity, but focused experiments are natural in the context of exploratory research, and in this case, they do not reflect an objective limitation regarding the use of human brain data. When we consider instead the full range of available neuroimaging datasets, and compare it with the semantic data usable for general-purpose foundation models, a more comprehensive picture emerges. According to recent estimates, the indexed web contains around 510 trillion tokens of text (Villalobos et al., 2024), which corresponds to approximately 2,000 terabytes of data. The total volume of neuroimaging data currently available is more difficult to evaluate, but NEMAR, a public platform for EEG and MEG data, has recently reported using a 200 terabyte storage system (Brandmeyer et al., 2026). At the time of writing, OpenNeuro, another public platform for neuroimaging data (Markiewicz et al., 2021), contains more than a thousand MRI repositories, which, considering the typical size of such repositories, could correspond to approximately 100 terabytes for this modality alone. Overall, in terms of order of magnitude, it seems reasonable to think that the volume of neuroimaging data currently available, across all public platforms and neuroimaging modalities, might not be substantially different from the volume of text data of the indexed web. Naturally, these volumes provide only an indirect estimate of the potential training corpora for both classical and brain-trained foundation models: for example, LLMs are typically trained only on a curated subset of the available text data, and neuroimaging datasets often contain anatomical images, which may be less valuable than functional data in the context of foundation model training. The objective of this quantitative comparison is not to imply that a text byte is directly comparable to a neuroimaging byte, but to highlight the full potential of human brain data as a possible large-scale training resource for AI. If the methods established for brain foundation models and multimodal neuroimaging research could be reused for the development of brain-trained foundation models, we speculate that a significant leap in scale and task diversity could follow, allowing us to gain a better understanding of the potential and limits of brain-trained AI. Moreover, such scaling would be perfectly compatible with the acquisition of dedicated neuroimaging datasets for strategically chosen, high-value steps in foundation model training, as these new datasets could be further prioritized for the extraction of particularly relevant or underrepresented neural signals.

Looking forward, if neural interfaces become more widely used in the future, potentially because of their seamless integration into wearable devices, some types of neuroimaging data could be acquired at scale in a diversity of real-life situations. This could result in a significant number of new neuroimaging datasets, perhaps noisier than in experimental settings, but possibly paired in real time with the stimuli, actions, or environments experienced by the neural interface users. Such datasets are unlikely to replace the neuroimaging data acquired in carefully designed neuroscience experiments, and they would not be a substitute for other measures such as fMRI or MEG, but they might allow for the extraction of relevant neural signals in a variety of realistic settings, and potentially expand the possibilities of brain-trained AI by reaching beyond the constraints and simplifications often inherent to experimental contexts. Another future opportunity for neuroscientists could be the possibility to use brain-trained foundation models as computational models of human cognition. Critically, existing research has already demonstrated that guiding deep learning models using neuroimaging data can provide insights into the cognitive processes of the human brain (Hansen et al., 2025). If such efforts can be scaled, or if neuroimaging data can be prioritized for strategically chosen steps, brain-trained foundation models could be repurposed by the neuroscience community as a new class of large-scale, open-ended computational models for simulating human cognition. In this sense, current frontiers such as leveraging neuroimaging data for alignment or reasoning could eventually unlock new possibilities for neuroscience. For example, if the reasoning capabilities of foundation models can be improved using neural signals from the prefrontal cortex, we speculate that such foundation models might offer new insights on human executive function in the context of long naturalistic tasks, by generalizing from the patterns acquired in shorter neuroimaging experiments.

## 5 A new role for neuroscientists

If human brain data lives up to its promises as a valuable and open-ended resource for AI, this could translate into a new role for the researchers responsible for acquiring and curating this resource. Neuroscientists are already experienced in evaluating which dimensions of human cognition should be measured, and they routinely compensate the limited scale of their individual neuroimaging datasets with sophisticated experimental designs that allow for a deeper understanding of cognitive processes. The transition from narrow AI systems to general-purpose foundation models such as LLMs, which often seem implicitly designed to function as an idealized form of human cognition, could gradually increase the relevance of neuroscience datasets, methods, and discoveries for addressing the remaining limitations of these models. In return, hypothetical brain-trained foundation models could serve as a new class of computational models for simulating human cognitive processes, therefore closing the loop. As a result, while pursuing their primary objective of understanding the human brain and other nervous systems, neuroscientists might also significantly shape the future of AI by selecting the populations and cognitive functions to investigate, the experimental designs to build, and the neuroimaging modalities to use, knowing that this contribution might eventually be beneficial to their primary endeavor. This new role for neuroscientists could remain relevant regardless of the specific algorithms or architectures that may become dominant in AI research, as long as the training strategies of the models can significantly benefit from the insights provided by neural signals.

Overall, brain-trained AI could offer an opportunity for neuroscientists to take on a new form of scientific leadership, and strengthen the impact from neuroscience to AI by providing the experimental resource, and not only the theoretical inspiration, for future foundation models. In return, this resource could help foundation models to evolve from an idealized form to a more neurally grounded approximation of human cognition, potentially unlocking new possibilities for the simulation of cognitive processes. Although the empirical results already available are encouraging, especially given the limited number of subjects included in some of the experiments, further research would be needed to better understand the full extent of the improvements that might be gained from neuroimaging data. While we should not assume that neuroimaging data will be a universal solution for overcoming all the limitations of foundation models, neither should we overlook the potential of this new strategy for AI, or its implications for neuroscientists, as all the semantic data on which current foundation models are trained is ultimately generated by the activity of human brains.

## Data Availability

The original contributions presented in the study are included in the article/supplementary material, further inquiries can be directed to the corresponding author.
